# Institutionalizing evidence-based practice: an organizational case study using a model of strategic change

**DOI:** 10.1186/1748-5908-4-78

**Published:** 2009-11-30

**Authors:** Cheryl B Stetler, Judith A Ritchie, Jo Rycroft-Malone, Alyce A Schultz, Martin P Charns

**Affiliations:** 1 Health Services Department, Boston University School of Public Health, Independent Consultant, 321 Middle St, Amherst, MA 01002, USA; 2 McGill University Health Centre & School of Nursing, McGill University, Montreal, Quebec, CA; 3 Centre for Health-Related Research, School of Healthcare Sciences, Bangor University, UK; 4 Alyce A Schultz and Associates, LLC, 5747 W Drake Court, Chandler, AZ 85226, USA; 5 VA HSR&D Center for Organization, Leadership and Management Research, Boston, MA, 02130 USA; 6 Health Policy and Management Department, Boston University School of Public Health, Boston, MA, 02118 USA

## Abstract

**Background:**

There is a general expectation within healthcare that organizations should use evidence-based practice (EBP) as an approach to improving the quality of care. However, challenges exist regarding how to make EBP a reality, particularly at an organizational level and as a routine, sustained aspect of professional practice.

**Methods:**

A mixed method explanatory case study was conducted to study context; *i.e*., in terms of the presence or absence of multiple, inter-related contextual elements and associated strategic approaches required for integrated, routine use of EBP ('institutionalization'). The Pettigrew *et al*. Content, Context, and Process model was used as the theoretical framework. Two sites in the US were purposively sampled to provide contrasting cases: *i.e*., a 'role model' site, widely recognized as demonstrating capacity to successfully implement and sustain EBP to a greater degree than others; and a 'beginner' site, self-perceived as early in the journey towards institutionalization.

**Results:**

The two sites were clearly different in terms of their organizational context, level of EBP activity, and degree of institutionalization. For example, the role model site had a pervasive, integrated presence of EBP versus a sporadic, isolated presence in the beginner site. Within the inner context of the role model site, there was also a combination of the Pettigrew and colleagues' receptive elements that, together, appeared to enhance its ability to effectively implement EBP-related change at multiple levels. In contrast, the beginner site, which had been involved for a few years in EBP-related efforts, had primarily non-receptive conditions in several contextual elements and a fairly low overall level of EBP receptivity. The beginner site thus appeared, at the time of data collection, to lack an integrated context to either support or facilitate the institutionalization of EBP.

**Conclusion:**

Our findings provide evidence of some of the key contextual elements that may require attention if institutionalization of EBP is to be realized. They also suggest the need for an integrated set of receptive contextual elements to achieve EBP institutionalization; and they further support the importance of specific interactions among these elements, including ways in which leadership affects other contextual elements positively or negatively.

## Background

Organizational context is receiving attention from researchers across multiple disciplines as a potential factor in the successful implementation of evidence into practice [1-5]. Although individual-level determinants of research use have received primary emphasis historically, findings from the fields of quality improvement (QI), research utilization (RU), and evidence-based practice (EBP) increasingly are demonstrating that a number of contextual factors may also play an influential role. More specifically, contextual factors at micro-, meso-, and macro-levels, such as leadership [6-10], culture and climate [11,12], access to resources [13,14], team climate [15], organizational slack [16], and organizational support [17,18] have emerged as potential mediators.

Despite this growing evidence base, we still do not know which contextual factors are more important, or how they operate or inter-relate to result in the successful implementation and use of evidence in practice. Furthermore, much of the existing research has been conducted with a focus on isolated practices or guideline and procedure-focused projects. There is little implementation research that focuses primarily on the overall context itself or, more specifically, on contextual factors related to institutionalization of EBP as a *routine *way of practicing (See definitions, Appendix 1). If one considers EBP institutionalization as an example of a strategic organizational transformation, then Harrison and Kimani's observations seem relevant to this knowledge gap [19]; *i.e*., 'accounts of transformation initiatives often reveal little about past organizational and contextual conditions that contributed to success. Instead, these accounts concentrate on change barriers.' While there are exceptions in the research literature [20,21], and pragmatic cases can be found where selected organizations are moving forward to routinize EBP [22-24], rarely are rigorous evaluations of related contextual and strategic processes presented. In summary, we know little about what specific set of contextual conditions interact to facilitate the institutionalization of EBP [25].

Against this background, there continue to be calls for more research. For example, there is a need to enhance our level of understanding of context sufficient both to guide organizational-level intervention studies as well as individual improvement/implementation practice change projects [1,11,26-28]. There is also a need to better understand configurations and the related combined presence or absence of contextual factors in relation to an organization's capacity to improve [29]. This paper presents the main findings from a case study addressing such gaps in the literature. Specifically, this theoretically-based study sought to identify key contextual elements and related configurations and relationships in an organization where EBP was perceived to be used routinely, in contrast to one in which it was not.

### Study purpose and framework

A published protocol [25] provides in-depth information about this study's background, theoretical framework and methods. This section of the paper provides a summary.

The study's primary research questions were:

1. What key contextual elements support and facilitate institutionalization, *i.e*., routine implementation of EBP and related projects, within a healthcare system at multiple institutional levels?

2. What strategic processes are used to create institutionalization of EBP within a healthcare system at multiple institutional levels?

The Content, Context, and Process model of the strategic management of change [30-35] was the study's theoretical framework. It has the following components: 'Elements' or signs and symptoms of receptivity related to more successful strategic change; and 'essential dimensions' of strategic change, *i.e*., the WHY/motivation for change, the HOW/process of change, and WHAT/content of change.

The framework also allows differentiation between a receptive and a non-receptive context. A receptive context has 'features ... (and also management action) that seem to be favourably associated with forward movement'; and a non-receptive context has 'a configuration of features which may be associated with blocks on change' [34].

## Methods

The study was a multi-method explanatory case study [36], with a core qualitative component and simultaneous supplementary quantitative component [37]. It focused on exploring the role and evolution of context in the routine use of evidence in practice within targeted services ('case'). A case was a department of nursing within a hospital.

### Sampling and recruitment

#### Sites

Two sites from different regions of the United States (US) were purposively selected to provide contrasting results for predictable reasons [36]. First, a 'role model' site was selected through a nomination process involving the American Organization of Nurse Executives (AONE) [25]; *i.e*., members of relevant AONE Boards were asked to identify '...widely recognized acute care hospital-based nursing departments that appear to have demonstrated the capacity to successfully implement and sustain EBPs to a greater degree than other nursing departments in the US ..., that is, nursing departments that appear to understand 'how to make EBP happen' and are seen as a role model by other nurse executives.' (See Additional File 1, 'Nomination panel letter for role case.') The selected department met the criteria of high ranking by the AONE panel; high self-rated level of institutionalization, with a brief substantiating rationale; and willingness to participate in the study and facilitate site access.

Second, a 'beginner' site was selected from AONE member volunteers self-reporting their department as 'early in the journey to institutionalization.' The selected site had low self-rated institutionalization, with a brief substantiating rationale, and willingness to participate in the study and facilitate site access. From among all volunteers, this site was a best match with the role model hospital's characteristics (Table 1).

**Table 1:** Chief characteristics of the case study sites

Characteristic	Role model site	Beginner site
Bed size	Over 350	Approximately 400

In-patient units	20	24

Type of hospital	Academic medical center	Community hospital (With multiple nursing school affiliations)

Chief nursing officer authority	Full administrative authority, with financial resources control	Full administrative authority, with financial resources control

Chief nursing officer type of position	A vice president of patient services in general, with responsibilities beyond nursing	A vice president of patient services in general, with responsibilities beyond nursing

Magnet status	Magnet designated hospital	Magnet application hospital

Other status	Non-Union	Non-Union

Self-perceived EBP status upon selection	More than three-fourths progress* along the scale toward full EBP integration Also self-reported: 'an intense focus on EBP'	Not even one-fifth progress along the scale* toward full EBP integration: Also self-reported: 'implemented some EBP initiatives... basic, nothing high level'

Case mix index, all payors	At the time of their site visit, both hospitals reported case mix indices in the low to medium intensity of resource use, with the role model site** reporting lower resource needs more similar to that of community hospitals, and the beginner site experiencing resource use suggesting moderate needs, higher than most community hospitals but lower than tertiary medical centers.	

Nursing education mix	The role model site had a very high proportion of BSN nurses, virtually double that of the beginner site.	

Hours per patient day (HPPD)	▪ Critical care: Last quarter (Jan-Mar 07) 19.8	▪ Critical care: 14.62
	▪ Med-surg: 9.92	▪ Med-surg: 5.22

*EBP Journey Scale

**START **- Starting to consider our EBP goals/vision ----- **END **- EBP is fully integrated into our structures and routines

**Role Model Site CMI: The role model site described a concern that their CMI did not reflect their level of patient acuity. After our study, the site had its CMI reassessed by DRG specialists and recently reported to us a new CMI, which is considerably higher than that used above and is now at a level consistent with their status as an academic medical center and their HPPD.

#### Site participants

Participants within each site were identified in two ways. Three embedded units within each site (medical/surgical, specialty, critical care) provided a pool of staff nurse participants. Second, within each site, a list of members of the hospital-wide nursing leadership/management team and other relevant EBP key informants was created by the site facilitator and local study sponsor, in collaboration with the principal investigator/PI (CBS). This list included both formal leaders, *i.e*., those in managerial positions at all levels of the hierarchy, and informal leaders, *i.e*., those in support/staff positions as well as other individuals perceived to influence EBP at either central or unit-based levels. Such informal leaders included educators, researchers, various specialists (such as clinical nurse specialists/CNSs, or QI resources), chairs or facilitators of EBP groups, and others viewed as 'leaders in EBP.' In particular, bedside nurses perceived to influence EBP, and thus defined in the study as informal EBP leaders, were sought. A purposively sampled set of all types of leaders was drawn from this list for individual interviews [25].

### Data collection methods

1. Individual interviews with leaders and focus group interviews with staff nurses: Interview questions were primarily developed within the framework's essential dimensions of the WHY, WHAT and HOW of strategic change [25].

2. Focused observations of pre-formed nursing and interdisciplinary groups relevant to EBP initiatives and naturally occurring at the time of the site visit, *e.g*., policy/procedure committee.

3. Document review of relevant EBP information, *e.g*., role descriptions [25].

4. Field notes from site visits by investigators.

5. Surveys including organizational learning survey/OLS for culture [38], multi-dimensional leader questionnaire/MLQ [39], nursing work index/practice environment survey/PES [40], and a research utilization (RU) tool [41], along with demographic information. Surveys were collated into a package and sent to all listed formal and informal leaders, as well as all staff nurses on the embedded units. Leaders were asked to focus their responses based on assessment of the chief nursing officer/CNO (MLQ), department as a whole (PES and OLS) or staff nurses as a whole (RU). Staff nurses were asked to focus their responses based on assessment of their unit (PES and OLS), nurse manager (NM)/ward sister (MLQ) or self behavior (RU).

### Analysis

#### Qualitative data analysis

Data were analyzed within site-specific data sets and then triangulated across site-specific data sets before making comparisons across sites. Analyses focused specifically on identifying content related to institutionalizing EBP.

**Figure 1:**
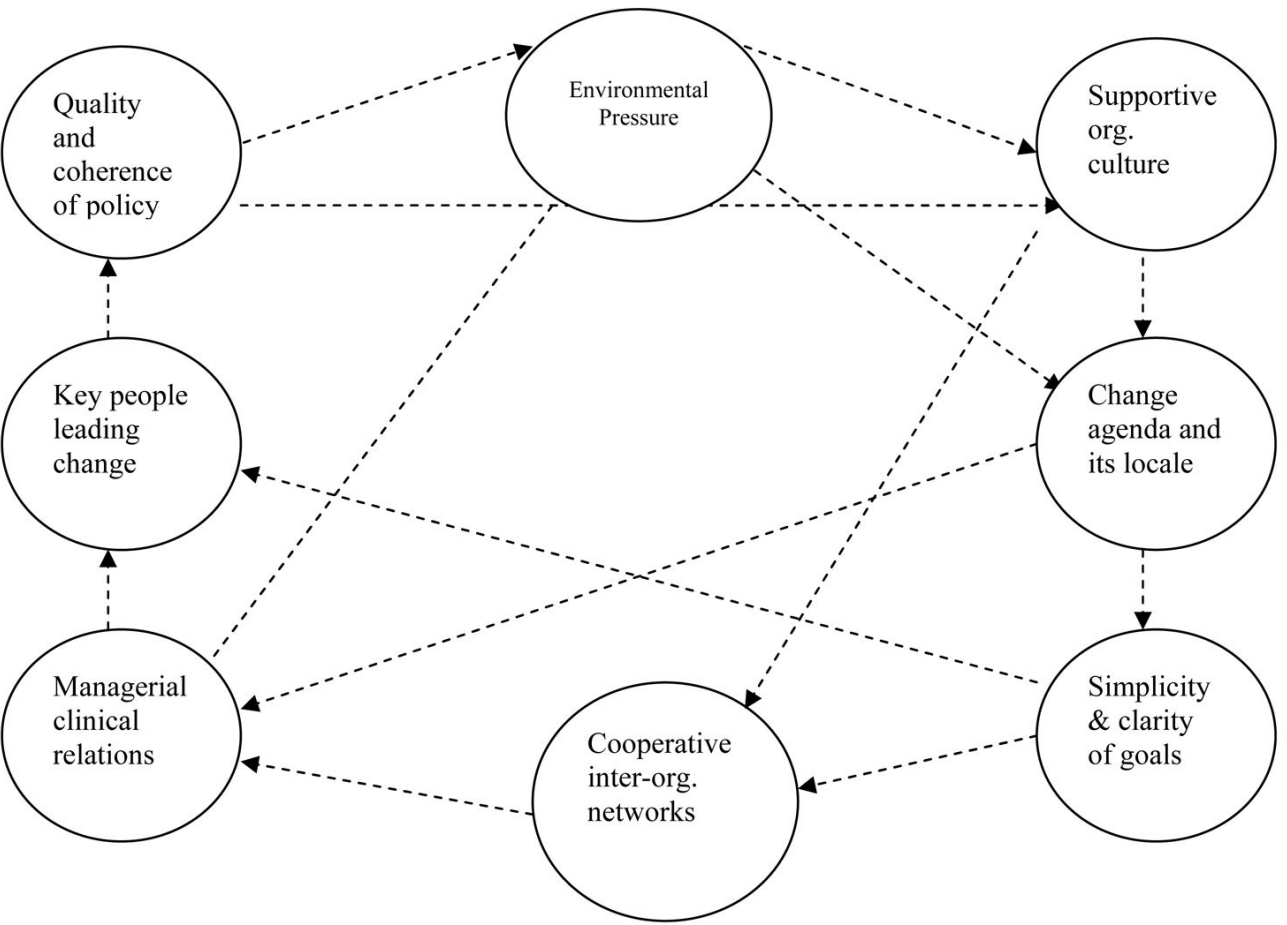
**Receptive contexts for change**. Reproduced with permission of Wiley-Blackwell: Pettigrew A, Ferlie E, McKee L: Shaping Strategic Change--The Case of the NHS in the 1980s. *Public Money & Management *1992, 12(3):27-31 (Figure 1, p 29).

An initial coding scheme was developed deductively based on basics of EBP change (*e.g*., definitions and barriers) and elements and dimensions in Pettigrew [33,34]. In terms of the latter, in addition to WHY, WHAT and HOW sub-categories under strategic management of essential dimensions, eight receptive elements (Figure 1) formed the basis for another major coding category (receptive context for change). This included sub-nodes for 'receptive' and 'non-receptive' content, per element. An inductive approach also was used to allow for creation of emerging codes. Data were managed in NVivo.

The role model site was coded first. This initial coding framework also applied to the beginner site data but required the addition of new sub-codes (*e.g*., Magnet and staffing). The PI took the main role in analysis, with other team members continuously checking/validating the approach and emerging findings. This often necessitated revisiting raw and coded data as well as clarifying and operationalizing definitions of contextual elements. The latter was needed as some of the framework's elements--culture, leadership, and coherence (Table 2)--did not have sufficiently clear definitions to enhance consistent coding decisions. Through this iterative team approach, agreement was reached on key findings and comparisons for each site. An audit trail was maintained throughout the analysis process.

**Table 2:** Elements of receptivity

**Pettigrew et al. elements **[34]	Study definition and observations
Change agenda and its locale	The element's focus is on the fit between the agenda and factors in the local, external environment that might influence internal change efforts.

Cooperative inter-organizational networks	Development and management of links with other agencies, *e.g*., through boundary spanners.

(Long term) Environmental pressure	The intensity and scale of pressures from influential agents external to the organization.

Key people leading change	• Defined by the team in terms of roles in which an individual influences others, more specifically, in terms of strategic versus operational influence, *i.e*., influencing others to behave in certain ways toward preconceived group goals (Schein) ___ in this case EBP in a department of nursing.
	• Types of roles were defined as formal, or managerial and related to positions of authority at all levels; or informal. Informal leaders included both clinical support personnel, such as APNs (Advance Practice Nurses) and special types of staff or EBP roles, either formal or informal.

Quality and coherence of policy	• The meaning of policy is broad, *e.g*., in the form of a broad vision, and not specifically about local policies and procedures.
	• More focused on strategic decisions relative to change, with quality referring to the related evidence base, related conceptual thinking about such decisions, and eventual buy-in
	• Coherence reflects initial exploration of a vision's congruence among related 'goals'; attention to politics and needed negotiation with key stakeholders; feasibility; and skill in terms of how the targeted strategic change was managed. In this study such congruence was defined as not only including development/refinement of organizational components on paper but the actual operationalization of such infrastructures for EBP; *i.e*., organizational structures, systems, roles, processes, relations, alignments, and capabilities.

Managerial-clinical relations	The quality of the interface between staff and management.

Simplicity and clarity of goals	• The ability 'to narrow the change agenda down into a set of key priorities, and to insulate this core from the constantly shifting short-term pressures' [34].
	• Demonstrates managerial '... persistence and patience in pursuit of objectives over a long period' [34].

Supportive organizational culture	Defined by the study team as the way things are done in an organization that is supported by its values, norms and expectations. Such forces in an organizational social system affect behavior of individuals.
	Culture can be characterized as strong or weak. In an organization with a strong culture there is high agreement among individuals regarding expectations and values, whereas the level of agreement regarding values and expectations is low or highly variable in a weak culture.
	Regarding EBP, values and expectations regarding use of evidence are direct aspects of a culture supporting evidence based practice. Related characteristics of a culture, such as values supporting collaboration and teamwork, are expected to support EBP.

#### Triangulation

Within the qualitative data analysis process, triangulation was used to refute or confirm emerging findings within each data set. For example, as leadership began to emerge as a key issue within interview data, this also was explored within focus group data and field notes.

Findings from our qualitative data helped provide a focus for what to report from survey data. For example, given leadership's emergence as a key qualitative finding, we were interested to investigate MLQ findings. In this way, triangulation provided us with a validation process, thereby increasing the trustworthiness of our findings.

#### Quantitative data analysis

Numeric data analysis was managed in SPSS, Version 15. Analysis of each survey instrument was conducted separately and followed the analysis procedures recommended by the originators. Two-tailed, independent sample t-tests were used to test mean differences between sites overall and between their leadership. Staff nurse samples were not compared statistically between sites due to their small size.

## Results

### Sampling

Table 3 provides a description of the 'sample' for each site, for each type of data collection. Greater participation was experienced in the role model site, despite the heavy work demands reported in both organizations. For example, at the role model site there were: proportionally more staff in focus groups and responding to surveys; more staff nurses who were identifiable as informal leaders, including special staff nurse roles relevant to EBP; and more groups with explicit links to EBP to observe.

**Table 3:** Summary of case site samples

SOURCES OF DATA	ROLE MODEL SITE N/TYPE PARTICIPANT	BEGINNER SITE N/TYPE PARTICIPANT
FOCUS GROUPS: on three units per case	Focus Group interviews = 9	Focus Group interviews = 5
• General med/surg unit; specialty unit; and a critical care unit.	Total staff nurse participants, multiple shifts = 27	Total staff nurse participants, multiple shifts = 14
• All staff, per unit, invited to one of several sessions.		

LEADERSHIP INTERVIEWS:	Total leadership interviews = 30	Total leadership interviews = 29
• Primarily formal leaders within nursing but also physicians, allied health and non-nursing top leaders.	Number of individual leaders = 26	Number of individual leaders = 28
• Informal leaders, primarily nursing	• FORMAL: 14	• FORMAL: 14
	- Top organizational leaders, *e.g*., chief nurse; her 'supervisor'; and chief MD	- Top organizational leaders, *e.g*., chief nurse; her 'supervisor'; and chief MD
	- Nursing clinical directors and nurse managers; and non-nurse clinical director and non-nurse manager, *e.g*., allied health	- Nursing clinical directors and nurse managers; and non-nurse clinical director and program leader, *e.g*., allied health
	- Nursing support or clinical resource services manager and non-nurse support service director	- Nursing support or clinical resource services manager and non-nurse support service director
	- Some also chairs of EBP-related committees/groups	- Some also chairs of EBP-related committees/groups
	• INFORMAL: 12	• INFORMAL: 14
	- Nursing support or clinical resource staff, such as researchers, APNs, or other various specialists relevant to EBP	- Nursing support or clinical resource staff, such as researcher or APN
	• Special staff nurse roles relevant to EBP on non-embedded units such as champion/facilitators or data/outcome specialists; some were also charge nurses	- Other various specialists relevant to EBP either within or outside of nursing, such as condition-specific educator or data/outcome specialists
		• Staff nurses involved in a special project or governance-related group; and an expert nurse

GROUP OBSERVATIONS	Groups = 5; Total participants = 74	Groups = 3; Total participants = 16
	• Policy/procedure-related and inter-disciplinary	• Policy/procedure and inter-disciplinary
	• Interdisciplinary clinical group	• Special QI group
	• Two special EBP groups, one interdisciplinary	• Nursing leadership group
	• Shared governance (PI invited)	

EBP-RELATED DOCUMENTS	• A multiplicity related to infrastructures, including, *e.g*.,	• Some related to infrastructures, including, *e.g*.,
	- Philosophy and mission	- Philosophy
	- More than a dozen on role descriptions and appraisal; clear focus in career ladder program	- A few nursing role descriptions; roles in QI department; included in career ladder program
	- Materials and minutes from multiple committees and interest groups heavily focused or specifically focused on EBP, some present for over five years	- A research group with materials, minutes and reference to EBP; QI groups, some clearly evidence-focused
	- Descriptions of governance groups, with EBP included in the expectations or activities of the majority	- Descriptions of governance groups, with EBP or data included in the expectations or activities of most
	- Educational and orientation materials, including EBP-related tools, presentations, skill sets	- Journal club material, PowerPoint presentation, and orientation description (*e.g*., re: library services)
	- Policy/procedure algorithm, researcher audit of related EBP status, and multiple Ps seen linked to evidence; clinical forms for documentation said to be E-B	- Policy/procedure algorithm, and Ps seen being linked to evidence; clinical documentation forms said to be E-B
	• Dozens related to EBP project activity and related dissemination efforts, internal and external:	• List of nursing research activity, including students and outside researchers; a PP hospital-based multidisciplinary project; a few single page PI outline for a improvement activities
	- Proposals for the human subjects committee decision	
	- PowerPoint (PP) presentations on EBP process and projects	
	- EBP-related project reports, program evaluations, and an EBP newsletter	
	- Publications, including multi-disciplinary ones; and evidence of co-operative networking	

SURVEY* FOR STAFF NURSES ON THREE EMBEDDED UNITS, with a focus on their unit or self	Respondents = 39	Respondents = 21
	Response rate = 34%	Response rate = 20%

SURVEY* FOR ALL IDENTIFIED MEMBERS OF THE LEADERSHIP TEAM, with a focus on the department	Respondents = 104	Respondents = 65
	Response rate = 56%	Response rate = 50%

*Tools in surveys: Organizational Learning, Multi-factor Leadership; Practice Environment; and Research Utilization.

### Overview of each case

#### 'Role model' case

Qualitative data showed that the role model site had been deliberatively and strategically building the capacity to successfully implement and institutionalize EBP over a period of more than five years. Within interview, focus group, field note, and document data, there was evidence of an approach that encompassed the essential dimensions of strategic change relative specifically to EBP. This included explicit attention to the WHY, or motivation/rationale for and enablers/barriers to strategic EBP change; the HOW, or methods of strategic EBP change; and the WHAT, or operationalized infrastructures of strategic EBP change [25] (Appendix 1).

Priority given to EBP at the role model site was evidenced through verbal communications and recurrent EBP language; a multiplicity of key documents, *e.g*., a vision/mission statement and role/performance expectations; a continuous record of nurse-initiated EBP projects and research, and ongoing, norm-related managerial initiatives (see EBP-related documents, Table 3). As one interviewee commented, 'EBP ... in your face every day but in a good way' (formal leader three). From an historical perspective, Magnet Recognition Program^® ^status (Appendix 1) was sought at basically the same time as the EBP effort was initiated. Further, the most influential, top EBP leaders were of long-standing tenure at the time of the site visit and had been present from the start or before the initiative; and visible progress and continuing, deep commitment to EBP were evident by years three to four.

#### 'Beginner' case

Qualitative data showed that the beginner site was a department in transition and at the time of the study visit, as initially self-reported, still early in the EBP institutionalization journey. Leaders in some cases felt they had made progress during the intervening period between selection and study visit. However, it should be noted that the so-called 'beginner's' focus on the Magnet Recognition Program^®^, which references EBP, was reported to have begun more than three years earlier; and although at the time of the visit there was evidence of a clear intent to build capacity to successfully implement EBP, most structural attempts--as noted in analysis of interview, focus group, field note, and document data--had yet to be adequately operationalized and thus realized as a routine, day-to-day activity. It is also of note that the two top leaders at the beginner site, comparable to the noted EBP influential leaders at the role model site, arrived after the initial Magnet work had begun.

EBP was rarely articulated by beginner site study participants as an ongoing explicit priority or vision. As one key leader noted, 'I don't think we have a clear vision and strategic plan for how we are going to use this.' Interviewer: 'In terms of EBP?' Key leader: 'Exactly. Exactly.' Instead, a clear priority at the time of data collection was achievement of 'Magnet' status (Appendix 1): 'We've been doing Magnet rounds for, I don't even know how long. We go on rounds to talk about Magnet, to answer any questions that they might have...' (informal leader thirteen). Outcomes were also designated as a clear priority, but again not in a way that was clearly connected to EBP. Overall, based on multiple sources of data, it was the judgment of the study team that the Magnet effort seemed to detract some key players from the EBP institutionalization aspect of the initiative, rather than reinforce it.

Further, data showed that some key leaders at the beginner site focused more heavily on the conduct of research rather than its use, which is consistent with the Magnet Recognition Program^®^. The department also tended to focus on an organization-wide priority of collecting QI audit and outcome data, which was heavily geared to externally defined performance indicators (*e.g*., from Centers for Medicare/Medicaid Services). Although intended to enhance quality, such data or related collection activities were perceived by multiple participants as problematic; *e.g*., '...there was all this data out there and I didn't know where it was coming from. And how it was collected. And what was the strength of this evidence; not evidence but data' (informal leader nine).

### A general cross-comparison between cases

The two cases were clearly different in terms of EBP relative to their organizational context, level of EBP activity, and degree of institutionalization. In general, the role mode site had a pervasive presence of EBP versus an isolated presence in the beginner site. Unlike the role model site, the beginner site had only a handful of isolated nursing-led EBP projects or research, some still in the developmental stage. Additionally, nursing at the beginner site seemed driven primarily by external demands, traditional QI, and physician-focused initiatives. This was in contrast to the role model site's focus on EBP-related staff-driven issues and professional practice improvements, in addition to external demands. Another distinction between the cases was the clear leadership role played by nursing in EBP activity at the role model organization; in contrast, the most EBP-knowledgeable individuals at the beginner organization were key physicians. Few in nursing at the beginner site appeared to have in-depth knowledge of the concept of EBP or its related processes.

Overall, little hard evidence existed that the beginner site's department of nursing was consistently applying evidence to practice according to our study definition; *i.e*., in terms of a clear search for and systematic use of research findings, as well as other evidence--but particularly research--to improve identified practices or processes within nursing. Evidence suggested that the site was still, on the whole, in the awareness/beginning stages of EBP, with a recurrent reference by site participants to 'beginning' or 'beginning shift' or 'a ways to go.'

**Figure 2:**
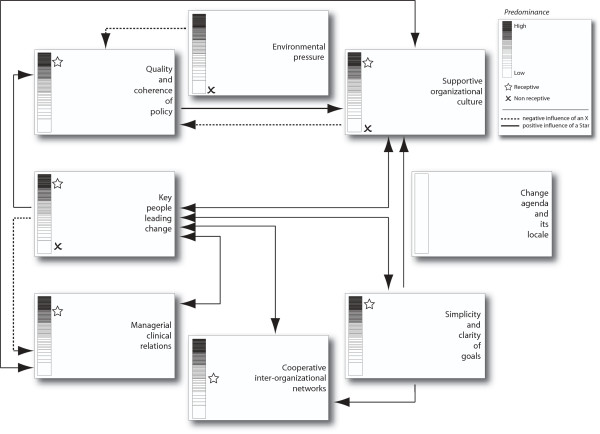
**Role model case**.

**Figure 3:**
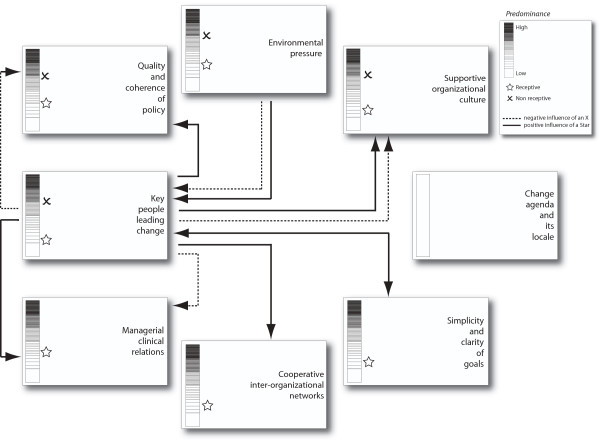
**Beginner case**.

In terms of the nature of their organizational context relative to EBP receptivity, the two sites were qualitatively different. More specifically, based upon accumulation of data from multiple sources and multiple participants, the team observed distinct differences in the extent or degree to which each case had progressed relative to its overall EBP receptivity in contrast to its overall EBP non-receptivity. In turn, the team qualitatively judged those differences on each of Pettigrew *et al*.'s individual elements [33,34]. While it was not possible to calculate quantitative scores, the team consistently agreed upon estimates of the general level of EBP-related receptivity and non-receptivity, per element, within each site. Figures 2 and 3 visualize these contrasting conditions with a vertical high-low scale to designate the predominance of receptivity and non-receptivity conditions.

The box in the upper right corner of each Figure contains the level or 'predominance' scale for receptivity/✰ and non-receptivity/✗, as well as the meaning of each type of symbol and arrow. A blank scale, as in the change agenda and its locale, indicates no discernible data regarding the presence and/or influence of that element at the site. The arrows, demonstrating element-to-element relationships, indicate either a positive or negative influence between specific elements as well as either a one-way or interactive relationship.

As indicated in Figures 2 and 3 overall, and as described in more detail in the following section, the role model site had a more discernible EBP-receptive context and a lower degree of non-receptivity than the beginner site. In contrast to the beginner site, the role model site demonstrated an interconnected combination of receptive contextual elements that appeared to enhance its ability to effectively and purposively institute and sustain EBP-related change. This included a greater number of more positively linked signs and symptoms/elements of receptivity in the role model site. In the beginner site, despite a positive intent and initial structural efforts, the elements of EBP-related receptivity were not yet operationalized to a sufficient degree to create institutionalization, with the site demonstrating a mixed or patchy context relative to strategic EBP change. Specifically, the beginner site presented a moderate to high level of non-receptivity in selected contextual elements, along with a fairly low level of EBP receptivity overall (Figure 3); and there was a greater number of, and stronger, negative linkages than in the role model site.

Statistically significant cross-case differences were also evident in all but one of the survey findings (Table 4). Both the overall and sub-scale scores of the PES [40] were significantly higher in the role model site. This is consistent with qualitative findings where the role model site's leadership, culture, and related staff attitudes were found to be more developed in terms of supporting EBP. Trend-wise, further examination indicated that staff in the role model site reported not only higher scores on the PES than staff in the beginner site, but also higher than leaders in the beginner site. In terms of the OLS, used as a proxy for a learning culture [38], the role model site scored significantly higher than the beginner site. This, too, is consistent with interview data and observations regarding a supportive culture.

**Table 4:** Survey results

COMPARISON:		OVERALL^@^			LEADERS ONLY	
**INSTRUMENT**		**Role Model** **Site**	**Beginner** **Site**		**Role Model** **Site**	**Beginner** **Site**

**MLQ**: Multifactor Leadership Questionnaire [39], Transformational Leadership Subscales^&^:0 to 4 scale	▪ Ideal attributes*	3.41	3.16	▪ Ideal attributes*	3.53	3.24
	▪ Ideal behavior*	3.26	3.04	▪ Ideal behavior	3.38	3.19
	▪ Inspirational motivation**	3.49	3.24	▪ Inspirational motivation*	3.58	3.34
	▪ Intellectual stimulation**	3.05	2.71	▪ Intellectual stimulation**	3.08	2.75
	▪ Individual consideration*	2.88	2.59	▪ Individual consideration	2.89	2.62

**NWI PES**: Practice Environment Scale of the Nursing Work Index [40]: 1 to 4 scale	Overall score***	3.20	2.85	Overall score***	3.23	2.89

**OLS**: Organizational Learning Survey [38]: 1 to 7 scale	Overall score**	4.73	4.38	Overall score*	4.86	4.60

**RU**: RESEARCH UTILIZATION[41]: 1 to 7 scale	Overall score	3.69	3.58	Overall score	3.74	3.55

^@^Total sample, including staff and leaders

^&^Most applicable subscale; further data available from PI

* p < .05 **p < .01 ***p < .001 one-tailed t-test

As measured by the MLQ for the CNO and NMs, both sites overall demonstrated transformational leadership. However, scores were significantly higher in the role model site and in the 60th to 70th percentile for four of the five subscales. For the beginner site, scores were in the 50^th ^to 60th percentile on three scores and the 30^th ^to 40th for two, including intellectual stimulation. This pattern is consistent with and reinforces the qualitative data regarding EBP, as transformational leaders define a vision, clearly communicate organizational values, and work to get cohesion among employees relative to organizational values and goals, in this case regarding EBP [42].

The remainder of the Results section below further contrasts the role model and beginner sites in terms of key themes of receptive capacity. Related details further illuminate the above general findings.

### Key contrasting themes

Themes that emerged for the most part relate to elements from the Pettigrew *et al*. framework [33,34]. Additional themes beyond that framework are described last.

#### Key people leading change

There were several key types of roles at multiple levels leading change in relation to EBP in the role model's nursing service; *e.g*., 'I feel that our practice is evidence-based or that our environment is evidence-based because of our leadership, from the CNO [to] having a lot of experts that are really and truly willing to help and support/facilitate those kinds of activities' (informal nurse leader four). Identified by study participants and the research team at the role model site, such key leaders included the CNO, research and education director, clinical directors, NMs, advanced practice nurses (APNs) and staff nurses.

For both cases the CNO was a key leader, but in a qualitatively different way. The CNO at the role model site, who worked very closely with the research and education director from the start of the effort, was viewed by participants as the key leader and driver of the strategic vision for EBP. As reported by both leaders and staff, this vision was clear and consistent over time, as was the day-to-day priority given to EBP by this leader. For example, '...we had a vision with the CNO and the [research and education director] to really move our nursing department into a situation where there's going to be realization of research, EBP' (informal leader two). This CNO furthermore, in collaboration with other leadership, 'stayed the course' of the EBP vision and its operationalization even through competing, major organizational change; and, at virtually the same time as EBP, initiated the Magnet journey and maintained an explicit priority on both.

The CNO at the beginner site also was identified most frequently as the key leader for change, as occasionally was the site's nurse researcher. However, such beginner site references to 'leading change' usually were not explicitly about EBP, but rather about the Magnet Recognition Program^® ^in general or the conduct of research. As one respondent suggested, the CNO 'has a lot of experience with Magnet; and that was one of the main reasons I think they brought her on board. So she's been really instrumental in pushing the institution to pursue this' (informal leader seven).

In contrast to the CNO role, operational actors such as clinical experts, APNs and NMs were consistently identified as influential EBP leaders *only *in the role model site. These actors had an impact at a different level, *i.e*., the unit/ward, through the following actions: providing day-to-day promotion, support, implementation and maintenance for EBP; acting as mentors to staff; and operationalizing the expectations of the CNO and managerial team.

Operational leaders in formal roles at all levels in the role model site actively engaged staff's participatory EBP involvement; *e.g*., 'I think [what helps make for our high level of EBP] is our [APNs] and managers. Our APNs are very forthright in coming up to nurses and saying, 'Hey, you know there's a good project for you, what do you think about running this or starting this project" (focus group one). This focus on active and expected staff involvement/empowerment is reflected in Figure 2 in the managerial-clinical relations element. It is also reflected in the significant number of staff in informal leadership roles leading EBP-related change. This included staff nurses who were encouraged and enabled to engage in EBP through special 'championing/facilitating' roles, special data/outcomes functions, and EBP project roles.

Within the beginner site, there were a few, relatively isolated people (*e.g*., a NM or clinical expert) within the nursing service described as specifically leading EBP. Those leading such change were doing so within localized EBP initiatives (*e.g*., regarding falls or other nurse-sensitive indicators), rather than within a broader mandate for strategically operationalizing EBP. In this site, it was difficult to identify informal leaders, with few staff nurses being described as leading EBP change.

#### A culture supportive of EBP

At the role model site, EBP was reported by leadership and staff to be engrained in the culture. EBP had become the norm or the 'way things are done' at all levels, and the culture was strongly focused on expectations of, and values related to EBP. Artifacts of such a culture were evident in documents (*e.g*., philosophy), processes (*e.g*., recruitment/interview practices), behaviors (*e.g*., CNO's EBP role modeling), structures (*e.g*., committees and funding mechanisms), and everyday language and discussions. For example, 'since I walked in the door it has always been one of the number one focuses or priorities that we always talk about, and you hear it in orientation, on the units, everyday, everywhere you go, that we talk about, EBP ' (informal leader one).

More broadly, there was a clear orientation at the role model site to *knowledge *and not just to the necessary tasks of clinical practice. An example of this culture of clinical inquiry/scholarship is as follows; 'I believe that the difference here is they ask a huge amount of questions; because they know they don't know something and they're trying to integrate the evidence and they expect and we expect that they will get the evidence' (formal leader one). Additionally, there was an orientation towards integration of improvement goals with EBP in everyday practices such that EBP became routine and was not just a set of isolated projects; *e.g*., 'What the CNO did was work on it [EBP] with the nursing staff, developing it and very slowly and gradually built [it] in as a culture. And then, I can't tell you for sure at what point in time, but you know this whole big initiative with quality and quality initiatives, you could really begin to see the tie between the two' (formal leader five).

In contrast, at the beginner site, the culture articulated by various participants can best be described as 'mixed' or in transition. Some participants articulated a new, desired culture, which included EBP, best practice, and a focus on outcomes; *e.g*., 'I would like to see it be the culture of the organization. That everything we do is based on EBP. And it's a constant...journey that we're taking as far as the bedside' (formal leader three). Additionally, there was evidence in some documents (*e.g*., model of care), some processes (*e.g*., budgeting), and some behaviors (*e.g*., a unit's positive project response) that isolated EBP or research efforts were making progress. There were also isolated units wherein EBP-related evolution was evident; *e.g*., where the NM--herself engaged in an EBP-related project--saw her role as a 'facilitator to make sure that we are constantly reassessing our standard of nursing care' through exploration of related evidence and 'an expectation of staff to figure out good clinical care. ... [and] ask questions and to wonder why' (formal leader four).

On the other hand, there was evidence that the beginner site's culture was more non-receptive than receptive (Figure 3). In particular, issues concerning accountability and slowness/resistance to change were articulated recurrently: 'And things move so slow... and other people have voiced that same sentiment around me so I don't think I'm isolated in my perspective' (informal leader 10); 'However, again because we've not been a culture that is very strong on accountability, if you don't hit that target again and again and again, what does that mean' (formal leader two).

Finally, there was a predominance of task-based nursing as opposed to knowledge orientation: 'We are a culture of task masters. I give the med at nine. I do the vital signs at ten. Oh, I've got to empty the Foley, so they empty the Foley' (formal leader two); 'They're doing what they're told by the doctor's orders. And they feel like that's, that's enough type thing [to do]...a lot of them' (informal leader seven).

#### Coherence of policy

The coherence of a policy/vision is achieved through the methods or the 'HOWs' of strategic change--in this instance, what key people leading change do beyond setting the vision/priorities and creating a supportive culture. Again there were key differences between the two cases. In the role model site, data show extensive, deliberate, and consistent activities and mechanisms to further integrate and institutionalize EBP over time. Such efforts were clearly based on an established 'policy' (Table 2), and primarily focused on the creation, or refinement of the WHATs, or departmental infrastructures, needed to integrate EBP into the fabric of the department's routine (Appendix 1).

As stated above, the role model site's CNO was instrumental in developing the EBP vision; but she and the research and education director additionally were instrumental in strategically making those expectations operational and in sustaining them. This was accomplished through planned and responsive infrastructure changes over time based on continual monitoring of EBP and implementation of needed refinements. A sample of the changes made is detailed in Table 5 as well as within 'documents' in Table 3.

**Table 5:** Sample infrastructures of strategic EBP change in nursing department

ROLE MODEL SITE	BEGINNER SITE
▪ Building EBP capacity (*e.g*., extensive orientation/education/skill development; EBP model review; active journal clubs; multiple research/EBP experts and mentors)	▪ Building mostly research capacity (*e.g*., some orientation/education; some journal clubs; a research expert)
▪ Providing enablers of EBP activity (*e.g*., internet resources; project funding; EBP-related councils)	▪ Providing enablers of activity (*e.g*., internet resources; research funding; a research champion)
▪ Creating special EBP-related roles and functions, including for staff nurses (*e.g*., facilitator/champions and data/outcome specialists)	▪ APN role created to enhance EBP/research**; a central 'EBP' role focusing on Magnet overall
▪ Creating broad-based EBP-related incentives and expectations (*e.g*., career ladders; clear performance expectations for roles and within governance structures)	▪ Creating incentives (*e.g*., career ladder and Magnet status)
▪ Integrating EBP into practice processes (*e.g*., policy/procedures and documentation).	▪ Integrating EBP into practice processes (*e.g*., policy/procedures and documentation)
	**NOTE: QI department has special roles that work collaboratively with nursing, particularly around performance indicators and hospital-wide initiatives; some expertise in EBP.

In one respect, the beginner site was similar to the role model site in that a number of EBP-related infrastructure activities and mechanisms were on paper and 'in progress' (Table 5; Table 3: 'documents'), albeit in many cases relative to conduct, not use, of research. For example, the development of evidence-based policies was progressing. However, the beginner site did not have integrated structures and processes to the same degree as the role model site; nor, in a number of cases, were their infrastructures actually operationalized to a significant extent, *e.g*.:

1. EBP was not clearly integrated within a spectrum of job descriptions and related evaluations within nursing.

2. The concept of journal clubs had been created as a means to routinely engage staff in EBP activities (or conduct of research); however, their existence was variable across units and, most frequently, they were neither existent nor well-integrated with other infrastructure changes. For example, as staff nurses in one focus group noted: nurse one: 'I think that's in the very beginning'; nurse three: 'I don't think we've actually had one'; nurse two: 'I've never heard of it' (focus group two).

3. The role of the unit-assigned APN, envisioned to routinely operationalize EBP at the unit level, was at an early stage of development; and although some APNs were engaged in EBP activities, they were not cited by participants as key EBP leaders.

4. The beginner site was experiencing challenges in engaging staff on the ground. While there was a push from the top for engagement through governance efforts, staff did not yet appear to be empowered or widely involved at various levels relative to EBP. This could be partly related to the fact that few EBP projects existed (Table 3), and to the concerns of bedside nurses regarding staffing (see below).

#### Non-receptivity

At the role model site, examples of non-receptivity were found, including identification of a scattered number of laggards who were resistant to change or not well-engaged in EBP (Figure 2). Additionally, there was evidence of a small number of non-receptive leadership or key people not leading change (Figure 2). A minority of formal leaders was reported as having a managerial focus that did not sufficiently include EBP. For example, there were instances where new NMs were not yet achieving competence with administrative skills--with a resultant inability to focus on EBP.

In contrast, the beginner site showed a moderate to high level of non-receptivity in several key contextual elements (Figure 3). Problematic cultural aspects and lack of operational infrastructures account for part of that non-receptivity. Also, 'leadership' was identified, to a significantly greater degree than in the role model site, as a barrier to EBP by participants from multiple levels of the organization: 'But I think that the system has been stuck. I think there's some managers that are clueless about EBP. It's just like, the way we're doing things, it's just because I said so' (formal leader four). Some barriers related to leadership indirectly, *e.g*., the existence of role confusion. In other instances, negative aspects of the culture were connected to leadership: (interviewer) 'What do you think the factors are related to lack of accountability?' (response) 'It is definitely the management, the leadership' (formal leader six). In terms of their prospective journey, however, 'healthy turnover' was noted by key beginner site leaders as part of the change process.

#### Environmental pressure

Although not a 'key' theme, the existence of 'negative' external environmental pressure relative to EBP (Figures 2 and 3) existed at both sites. At the beginner site, there was a strong focus on data collection linked to multiple regulatory and prominent benchmark pressures. This effort appeared to detract from other EBP activities, given that the high level of resources devoted to data collection was not always perceived as useful. These pressures were not a major issue at the role model site. However, there was a discernible concern at the latter regarding the growing demand from external agencies; *i.e*., in that such pressure could increasingly impact the ability of key leaders to develop and support staff as facilitator/champions of EBP. At the beginner site, there was also a positive environmental pressure; *i.e*., the professional value within the US of the Magnet Recognition Program^®^.

#### Other themes

Two other themes emerged inductively from the evidence: differential aspects of the cases' internal nursing and hospital environment as well as barriers to EBP and its institutionalization. These relate to the 'WHY' of change in terms of its enablers/barriers.

In terms of barriers, the role model site was struggling with a competing priority for time and attention at the organizational level, unrelated to EBP. This priority was absorbing an extraordinary amount of time, and individuals were struggling to maintain various EBP activities. However, the sense was communicated that, although 'it's unfortunate ... [as] I have a number of people who really want to do some projects, but just can't seem to get together and meet. ... At this point we need to get this [interim organizational priority] done so we can get on with business. ..., [and then] I think once that happens we're going to be in a good situation' (informal leader two).

A very different barrier was identified by nurses at multiple levels at the beginner site; *i.e*., lack of resources for EBP, research, education/practice, and related, knowledgeable experts at the unit level; 'Show me how it's really going to better the patient and myself and, again, that visibility and someone who is approachable every day...and not that the people that are in place aren't helpful, it's again, probably having enough--availability of experts' (formal leader seven).

In terms of the internal nursing and hospital environment, in the beginner site, staffing was viewed differently by staff versus key leaders. Staff nurses recurrently expressed a lack of sufficient staffing resources while some key leaders felt otherwise. In addition, interview data from various interviewees from all levels, except NMs, indicated a concern with poor practice; and in a few instances, interviewees reported multiple concerns. These concerns often related to the basics of nursing practice or ongoing lack of adherence to good standards; and at times, concerns related to staffing, lack of accountability or staff's lack of basic knowledge, particularly new graduates.

None of the above barriers (lack of resources, dissonance regarding staffing, or perception of poor practice) were noted at the role model site. Additionally, as described in Table 1, the role model site had considerably more staffing than the beginner site (measured by hours per patient day) and yet reported a lower case mix index (CMI). During the site visit, however, leaders at the role model site strongly argued that their reported CMI was inaccurate and did not reflect their actual level of acuity. Shortly after the site visit, the role model site reported to us that the CMI had been reassessed, by DRG specialists, at a considerably higher level (Table 1). In addition, relative to resources, the role model site had a preponderance of baccalaureate prepared nurses (BSN). Both the staffing and BSN statistics are suggestive of differences critical to EBP institutionalization, but insufficient data exist in this case study to fully clarify these relationships.

## Discussion

Two sites were sampled to provide contrasting results for predicable reasons. As predicated, findings showed a difference between sites with respect to institutionalization of EBP and corresponding contextual features. However, findings also provided theoretically-related, new insights regarding those differences.

While data were collected at a particular point in time, participants were able to provide historical and contemporary insights about EBP within their organizations. These resulted in a rich picture of their journeys to date. By using a strategic model of change [30-35] to compare sites, what emerged were 'key' contextual elements, the nature of those elements, and key inter-relationships that appeared to facilitate the ongoing and integrated use of evidence in practice--and thus may be critical to EBP institutionalization efforts.

Given those patterns of positive connections between key elements (Figures 2 and 3), the most influential element that appeared to affect the institutionalization of EBP was that of multiple, key people leading change. Within the role model site, this referred to people in both formal and informal leadership positions, at all levels of the institution, including bedside nurses.

### Leadership

In nursing, models to enhance the uptake of evidence into practice have been available for a more than a decade, *e.g*., the Iowa, Ottawa and Stetler models [43-45]. Such EBP frameworks are now referenced along with other implementation models (*e.g*., Grol *et al*. [46]) as 'planned action theories' that focus on steps needed to 'engineer change' for a focused EBP practice [47]. Although leadership may generally be recognized as important to such uptake processes, the concept of leadership does not appear as a core element in related visuals of these long-standing models. Nor do the above implementation models focus on the role of leadership in institutionalization of EBP as a routine.

Newer models regarding EBP or the more general concept of innovation in healthcare, however, are explicitly highlighting leadership. Some continue to focus on leadership relative primarily to an individual innovation [2], but others are beginning to reference leadership more broadly, for example in terms of: holistic conditions or ingredients for change needed for 'successful implementation' [48]; the context needed for strategic change or transformation in healthcare [32,33,49]; and institutionalization of EBP as a routine norm for practice [50].

Emerging research is also providing evidence regarding the influence of leadership at multiple levels on sustained use of research evidence in practice and related organizational change [7,8,26,27,49,51,52]. Our findings support such models and cited EBP research, which suggests that multi-leveled leadership by managers, educators, senior leaders, staff nurses, APNs, and supervisors characterizes organizations that have successfully implemented evidence into practice [8,53,54]. Additionally, as our research also found, an integrated approach coordinated by these leaders towards a common vision and goal appears to be key [8,49,54].

As Gifford and Davies [54] point out, there is a debate in the literature about the differentiation between leadership and management. They state it is likely that both effective leadership and skilled management are necessary to effect change in nursing practice [8,54]. Indeed our research, both in the qualitative and MLQ data, suggests that both leadership behaviors and management practices had a key role to play in creating a context receptive to EBP in the role model site, relative to an integrated set of receptive elements. Specifically, key leadership behaviors in the role model site included creating and sustaining a clear vision, role modeling, developing supportive relationships, and mentorship. Their management practices also went beyond a focus on isolated projects and included embedding/integrating EBP into the fabric of the organization through building structures, providing resources, monitoring progress, providing feedback, and changing formal leaders who didn't 'fit' with the strategic change/vision.

To realize the sustained, routine, and integrated use of evidence in daily nursing practice, our findings have implications for the development of formal leadership and management capacity, as well as for the development of capability within the nursing workforce for informal leadership. However, such findings and the role of key people leading change are not confined to nursing. Other disciplines and healthcare organizations appear to have the same challenges and needs relative to EBP, innovation, or strategic service change [5,49]. For example, Lukas *et al*. [49] found that leadership from top to bottom of an organization is a critical 'driver' of strategic organizational change focused on improvement of clinical quality.

### Culture

A number of scholars have suggested that culture is a contextual mediator of EBP and related strategic service change [20,48-50]. However, to date there has been little empirical evidence to support these assertions. Pepler *et al*. [12], through case study research, found culture to be a key factor in the use of research evidence within clinical units. They nevertheless found it difficult to disentangle the elements of culture in various units such that identifying one particular configuration of a culture that enabled research use was not possible. Lukas *et al*. [49] also cite culture as a key organizational component in sustainability of organization transformation. Our findings suggest that organizational culture is a contextual determinant of EBP institutionalization. As such, we argue that strategic leadership behaviors and management practices have the potential to create a culture of critical enquiry and scholarship, as observed at the role model site, in which EBP can become institutionalized.

### Inter-related elements

As noted above, we found a pattern of positive connections between key contextual elements in the role model site. However, this overall pattern of connections (Figure 2) is different from that found by Pettigrew *et al*. in their receptive sites (Figure 1) [33,34]. Additionally, we did not find the change agenda/locale element to be relevant, nor was the environmental pressure (Table 2) as significant as in Pettigrew *et al*. [33,34]. Newton *et al*. [5], in an exploration of a stalled change effort in a general medical practice, had similar findings for the latter two elements. However, they had yet another pattern of connections.

Pettigrew *et al*. [33,34] suggest that the receptive elements dynamically link and 'form a pattern receptive to desired change or innovation' but without a 'common, exact path or recipe by which these common factors come together to create success' [25]. Given different organizations and healthcare professionals, patterns might naturally vary. So too might some of the elements when the target of change varies or is as broad as institutionalization of EBP. In any case, the relevance of various connections remains unclear and needs further research to better understand whether they are significant, and if certain linkages create greater potential for success. Nonetheless, the involvement of leadership in those linkages, positive or negative, appears to be critical to overall receptivity or non-receptivity.

In summary, our research presented a 'snap shot' in time of both sites, albeit tapping into an historical context. In future research, tracking contexts over time would be useful; *i.e*., by taking a longitudinal perspective it would be possible to capture some of the complexities and dynamics occurring in contexts during the process of successful or unsuccessful EBP transformation [49]. In the meantime, the Pettigrew *et al*. model, in the context of the lessons learned in this study and other cited literature, may provide a useful lens for strategizing transformational efforts related specifically to EBP institutionalization.

### Limitations

While this study provides a comprehensive, in-depth picture of the potential influences of context on the routine use of evidence in practice, findings should be considered in the context of its limitations.

The study included only two sites. Therefore a consideration should be made of the findings' theoretical transferability to other contexts, rather than their generalizability. It is also possible that participants provided socially desirable responses. Potential threats to credibility were limited by data triangulation and the intensive period of time spent by the research team in the field. Additionally, the team was cognizant of the potential for 'leading' questions and took pains to review questions posed in the transcripts along with related types of participant responses. The team members routinely focused on affirmation of coding; and interview transcripts showed that participants generally provided balanced and open accounts. Overall, it was the team's judgment that interviewees were not unduly influenced nor coding selectively interpreted.

Although the study was limited in its historical data, and we could not pinpoint timing of various events precisely, the study team did obtain a clear sense of a continuing, strategic progression in the role model site over a period of more than five years. Data from multiple key leaders at the role model site provided a clear and consistent sense of the type of strategic decision making that occurred--given the site's vision and goal of continuous improvement toward that vision. Such EBP-related strategic progression was not found at the 'beginner' site. The team's ability to obtain historical information was more limited at the latter site, in part due to the tenure of some of the newer, key players and the lack of time and resources to pursue various 'historians.'

## Summary

Our findings provide evidence of some of the key contextual elements that may require attention if the institutionalization of EBP is to be realized. The most critical element in this study appeared to be key people leading change, which in turn impacted on the operationalization of other key elements of the strategic change model. A number of propositions have been developed from our findings, as follows:

• Organizations that achieve a highly receptive context for EBP, as described by Pettigrew *et al*., are more likely to exhibit a higher level of EBP institutionalization.

• Organizations with elements of receptivity (as described by Pettigrew *et al*.) and that monitor and act on elements of non-receptivity are more likely to exhibit a higher level of EBP institutionalization.

• Efforts to transform an organization for institutionalizing EBP requires the proactive, meaningful engagement of formal and informal leaders at all levels of the organization, including staff nurses.

• A greater number of positive two-way inter-connections between key people leading change and other key contextual elements in the Pettigrew framework will enhance an organization's potential for institutionalization.

• An organization with a majority of BSN staff nurses and competent, EBP-oriented nurse/ward managers will exhibit greater integration of EBP in routine practice.

• Executive leaders who have the ability to proactively influence an organization's culture to support EBP and can buffer the related strategic vision from periodic pressures are more likely to institutionalize EBP over time.

• Inconsistent operationalization of EBP-related infrastructures (coherence in the Pettigrew framework) by formal leaders will negatively impact an organization's ability to institutionalize EBP.

• Organizations that develop a strategic plan to institutionalize EBP using Pettigrew's key contextual elements as a foundation for professional practice are more likely to have a higher level of EBP activity within three to five years.

These propositions could be tested in future research and/or considered by those embarking on the institutionalization of EBP. Importantly, our findings indicate that there are a number of contextual factors that are modifiable; and they also show that related modification requires strategic intent and operational follow-through, with changes continuously monitored and sustained over time.

In conclusion, our findings regarding individual organizational elements such as leadership may not appear to be new. However, there remains a need to increase our knowledge both about the elements that overall and together support EBP institutionalization (or even use of EBP) and about the ways such factors influence achievement of these goals. What specifically is new from the study and needs further theory-based affirmation or clarifications are the following: The apparent need for an identified *set *of receptive contextual elements to achieve EBP institutionalization; the observed interaction among identified elements, including the ways in which leadership affects other elements positively or negatively; use of the Pettigrew *et al*. framework to guide EBP institutionalization/research; focus on the concept of non-receptivity, which is seldom found in the literature; and the way in which a nursing department achieved institutionalization through use of strategic actions (the HOW) in relation to key receptive elements (the WHATs).

## Competing interests

The authors declare that they have no competing interests.

## Authors' contributions

All of the authors take responsibility for the findings reported in this work and have read and approved the final manuscript. CBS took the main role in implementation of the study plan, including site visits, analysis, and drafting of the manuscript. All other authors (JR, JR-M, AS, and MC) actively participated in analysis of data, interpretation of data, revision of the manuscript, and support of overall implementation. JR and AS briefly participated in the site visits The home sites of investigators (JR, JR-M, AS, and MC/CBS) contributed not only the anticipated in-kind support but in two cases (JR and MC/CBS) provided extensive additional resources to enable completion of the project. See protocol [25] for additional contributions.

## Appendix 1. Refined study definitions

• **Context/organizational context**:

◦ Overall: The healthcare environment in which practice takes place; characterized by organizational culture, leadership, basic organizational components, and type of clinical setting.

◦ Pettigrew/Whipp: an essential dimension or the **WHY**/motivation behind strategic change to EBP and related enablers/barriers.

• **Content**: One of Pettigrew/Whipp's essential dimensions, in this case the **WHAT **of strategic change; *i.e*., the organizational elements or processes in the system changed to enhance or support the use of evidence.

• **Evidence-based practice (EBP)**: Practice derived from the best available evidence to achieve positive outcomes; this practice may range on a continuum from implementing a discrete practice (*e.g*., consistently using an evidence-based scale to assess the situation and implementing research-based interventions) to consistent ways or patterns of decision-making and practice (*e.g*., consistently seeking the best evidence in all decision-making to achieve positive outcomes).

• **Evidence**: Knowledge derived from a variety of sources that has been subject to testing and has been found to be credible. This includes:

◦ Research,

◦ Patient experiences and preferences, and

◦ Practical knowledge and local data (*e.g*., audit, quality assessments, planning and project data)

• **Infrastructure**: Organizational structures, systems, roles, processes, relations, alignments, and capabilities.

• **Institutionalization**: Integration of EBP into the routine fabric of the organization [50]; also known as **institutionalization**.

• **Levels within the institution/institution levels**: Individual, group/team, organization, larger external system. In this study, these levels refer to individual clinicians and leaders; EBP-related project teams or committees; clinical units; clusters of units within a service; department of nursing; hospital; and external healthcare-related environment.

• **Magnet status**: The Magnet Recognition Program for Excellence in Nursing Services^®^, provided by the American Nurses Credentialing Center (ANCC), recognizes outstanding healthcare facilities and systems that demonstrate excellence in patient care and work environments that attract and retain nurses, primarily in the US. Facilities are evaluated on their excellence in nursing leadership, shared governance, staff decision-making, the generation of new knowledge through nursing research, and the use of best evidence to support nursing practices and improve patient outcomes http://www.nursecredentialing.org/Magnet.aspx. Magnet has 14 forces; *i.e*., quality of nursing leadership, organizational structure, management style, personnel policies and programs, professional models of care, quality of care, quality improvement, consultation and resources, autonomy, community and the healthcare organization, nurses as teachers, image of nursing, interdisciplinary relationships, professional development. Expectations for the use of evidence are threaded (integrated) throughout the forces.

• **Non-receptive context for change**: 'A configuration of features which may be associated with blocks on change' [34].

• **Norm or routine per EBP**: Integrated into the everyday work of the clinical setting, in the policies, in the practices, in documentation, in the infrastructure, etc.

• **Nurse manager**: The leader on a particular patient care unit/ward. Such a role has direct responsibility and accountability for one to two clinical units or wards in terms of budget, hiring, firing, evaluation, quality, and daily operations.

• **Process**: One of Pettigrew/Whipp's essential dimensions [30], in this case the **HOW **of strategic change; *i.e*., the methods, strategies, or implementation interventions used to try to enable the use of evidence.

• **Receptive context for change**: 'A combination of factors from both the inner and outer context that together determine an organization's ability to respond effectively and purposively to change [2].

• **Strategic**: Refers to planned, organizational approaches to change and its deliberate management.

• **Sustainability**: Changes (practice and outcomes) based on evidence that continue over time as related to specific projects.

## Supplementary Material

Additional file 1 Nomination panel letter for role case. Click here for file
