# Supplementary material for: Institutionalizing evidence-based practice: an organizational case study using a model of strategic change
Source: Implement Sci. 2009 Nov 30;4:78. doi: 10.1186/1748-5908-4-78 (PMC2795741; doi:10.1186/1748-5908-4-78)
Supplement: Additional file 1 — Nomination panel letter for role case. [file 1748-5908-4-78-S1.PDF]

(Panel letter for selection of role model case)

Dear _______________,

As a member of the AONE Board and/or Institute for Patient Care Research & Education we are contacting you about our RWJ funded study on *Improving the Quality of Care through Routine, Successful Implementation of Evidence-based Practice at the Bedside: How Does It Happen?* A full abstract of the study, which as you may know was endorsed by AONE and the Institute, is inserted below for your information.


Evolving science in the area of evidence-based practice (EBP) supports the critical role of context, i.e., the critical role of the health care environment in which practice and EBP efforts take place. Despite this evolving knowledge, it is unclear exactly WHAT key contextual elements are needed and HOW executives and other organizational leadership can achieve this contextual quality. This project will study the role and evolution of context in the routine or on-going translation of evidence into practice within targeted services. The "targeted service" in this study will be departments of nursing, a critical player in quality in any health care organization.

This is a multi-method/multi-source explanatory case study, structured around the theoretical framework of Pettigrew and Whipp relative to the strategic management of change. This theoretical model has been widely used to analyze and retrospectively learn from organizational change programs. It was based on empirical case-based organizational research, including health care, and focuses data collection in three areas: the WHY of strategic change, the WHAT of strategic change, and the HOW of strategic change. In this case the strategic change is related to the implementation and institutionalization of evidence-based practice (i.e., integration of EBP into the routine fabric of the organization; also known as normalization).

A case study approach is the method of choice to address this topic, given the complexity of organizational phenomena. This method will provide an exemplar of the WHY, WHAT and HOW of a case known for a high level of EBP; i.e., a hospital that is widely recognized to have a nursing service using a deliberate approach to build the capacity to successfully implement, sustain, and 'normalize' EBP activity. This case will be contrasted with one that is just beginning the journey to institutionalization. The study will also provide clarifying information about the potential usefulness of three EBP models designed to provide guidance to nurse executives and other leadership on how to achieve successful implementation.


Our first task is to identify, through a systematic process, those acute care hospitals that are *widely recognized to have a nursing service using a deliberate approach to build the capacity to successfully implement, sustain, and 'normalize' EBP activity.* To do this task, we need your assistance as a member of a knowledgeable group of CNOs in the US. Specifically, could you please do the following:

1. Please list up to 5 widely recognized acute care hospital-based nursing services that appear to have *demonstrated the capacity to successfully implement and sustain evidence-based practices to a greater degree than other nursing services* in the US … that is, nursing services that appear to understand "how to make evidence-based practice happen" and are seen as a *role model* by other nurse executives. Use the longer lines below for your response. You may identify your own institution, if applicable.

    i. ____ ______________________________________________
    ii. ____ ______________________________________________
    iii. ____ ______________________________________________
    iv. ____ ______________________________________________
    v. ____ ______________________________________________

2. Please rank order your above listing of hospitals, beginning with the most successful role model as #1. You may use the same ranking for equally successful hospitals.

3. Please write a rationale that explains your nomination of these top nursing departments relative to EBP.

4. Please choose the option/s below that best describe *your* current position or one you have held within the past 5 years (This allows us to describe the "panel" that helped to nominate the "role model" case):

   - Type of position (check one):
     1. _____ Chief Nurse Executive, with administrative authority
     2. _____ Chief Nurse Executive, without administrative authority
     3. _____ Other position (please describe)

   - Type of hospital where position held (check all that apply):
     1. _____ Academic medical center
     2. _____ Teaching hospital
     3. _____ Community hospital
     4. _____ Magnet designated hospital
     5. _____ Rural hospital
     6. _____ Other key descriptor =

   - Size:
     1. _____ Number of beds
     2. _____ Number of nursing units

Please return this form either electronically to cheryl.stetler@the-spa.com or by fax to 413-253-3841 by ______________________.

Thank you for your assistance. Once nominating data have been collected from the AONE Board and/or Institute, the research team will identify potential cases that fit our criteria and, after discussion with the related CNOs regarding interest and feasibility, a selection will be made. Your participation has been invaluable.

Thank you, on behalf of the study team,

Cheryl B. Stetler, PhD, RN, FAAN
Principal Investigator
